# Decoding Hepatic Portal Venous Gas: A Case Report

**DOI:** 10.7759/cureus.54050

**Published:** 2024-02-12

**Authors:** Iulian A Dogaru, Daniela E Gheoca Mutu, Bogdan M Ursuț, Florin M Filipoiu, Adrian D Tulin

**Affiliations:** 1 Discipline of Anatomy, Carol Davila University of Medicine and Pharmacy, Bucharest, ROU; 2 Clinical Department of General Surgery, Prof. Dr. Agrippa Ionescu Clinical Emergency Hospital, Bucharest, ROU; 3 Clinical Department of Plastic and Aesthetic Surgery and Reconstructive Microsurgery, Prof. Dr. Agrippa Ionescu Clinical Emergency Hospital, Bucharest, ROU

**Keywords:** small bowel pneumatosis, mesorectum, portal venous system, paralytic ileus, hepatic portal venous gas

## Abstract

Hepatic portal venous gas (HPVG) is an infrequent and life-threatening condition with high morbidity and mortality rates, which consists of the presence of gas in the portal vein and its branches. Improvements in imaging technologies have led to the diagnosis of HPVG in less severe circumstances, which, in turn, has only determined a small amelioration of the prognosis. We present a rare case of HPVG subsequent to paralytic ileus in a patient who attained long-term survival after the surgical treatment was performed. HPVG is considered to be associated with sepsis, parietal/mucosal damage, inflammation of the intraperitoneal organs, and meteorism, which may be found in a variety of pathologies. The severity of this pathology depends on the pre-existing conditions of the patients but also on how quickly a treatment plan is established and applied. As a correct and timely diagnosis is crucial for the increase of the survival rate in HPVG, greater attention shall be paid to the clinical manifestations and the differential diagnosis.

## Introduction

Hepatic portal venous gas (HPVG) is an infrequent and ominous pathology, which consists of the presence of gas in the portal vein and its branches. It was first noted by Wolfe and Evans in 1955 in a radiological examination of six newborn babies who suffered from necrotizing enterocolitis [1]. HPVG is known to be associated with four main pathophysiological factors: the damage of the walls of the gastrointestinal (GI) tract (most importantly, the mucosa), the air distention of the bowel, intra-abdominal inflammatory processes, and the occurrence of sepsis [2,3]. The gravity of this condition is given not only by the mere presence of gas in the portal vein but also by the subjacent pathologies that lead to the passage of air from the intestinal lumen to the portal venous system. Of these conditions, the one that poses the highest vital risk is intestinal ischemia, which has a mortality rate of 75% [4]. Advances in the imaging techniques used nowadays have led to the diagnosis of HPVG in less severe circumstances, which, in turn, has only determined a slight decrease in the mortality rate [5,6].

## Case presentation

We report the case of a 73-year-old diabetic male patient who presented to the emergency room of "Agrippa Ionescu" Clinical Emergency Hospital with a one-month history of rectorrhagia episodes and pain in the anal region, with no other related symptoms.

The patient underwent a colonoscopy, which revealed a 15 cm long, friable tumoral mass, with the inferior limit at the level of the pectinate line, from which multiple biopsies were collected. The histopathological examination of the biopsies concluded that the tumoral mass was an intestinal-type adenocarcinoma (ITAC), which infiltrated into the mucosal layer. A computed tomography (CT) scan was also performed, and it showed a vegetant tumoral mass located at the level of the rectum.

**Figure 1:**
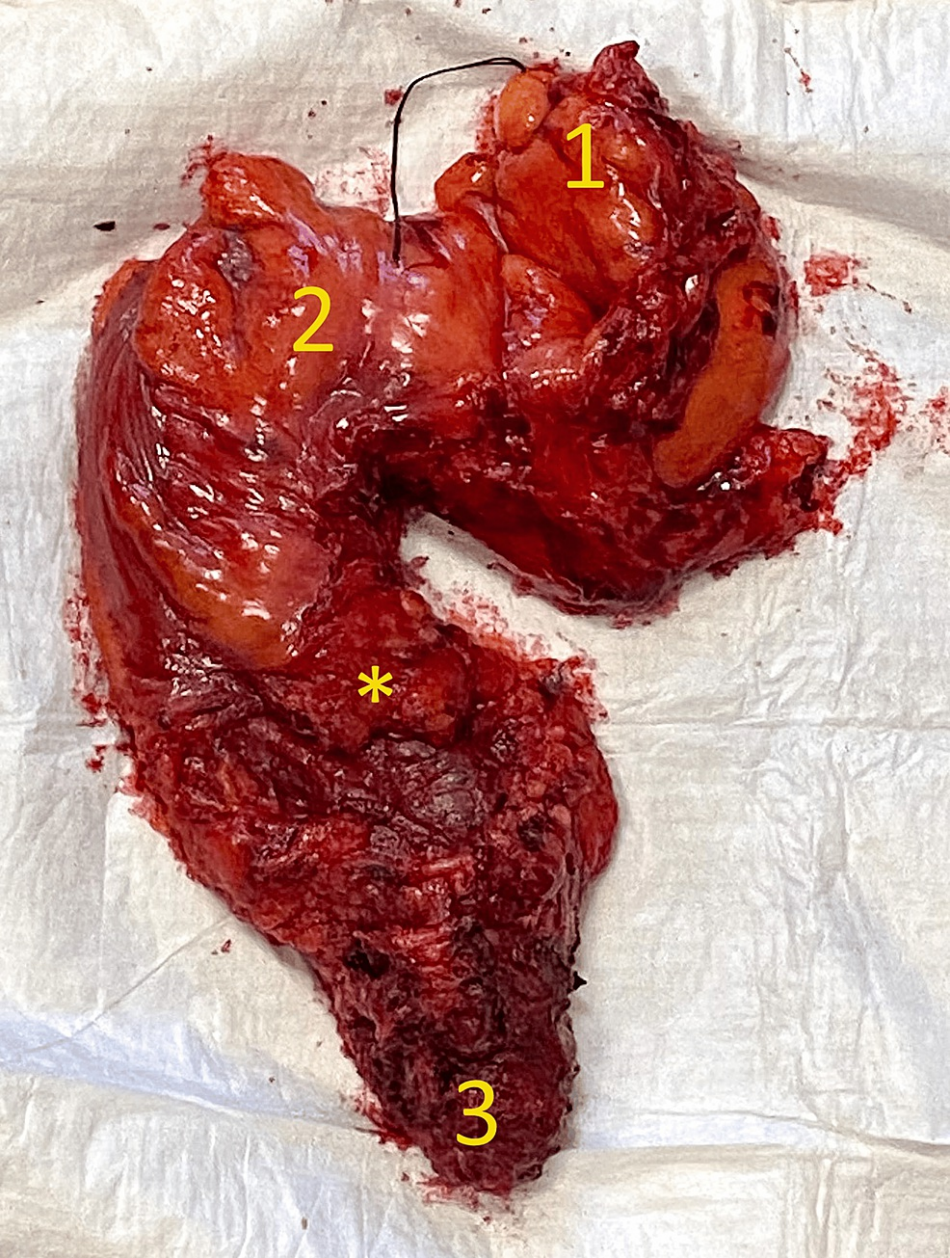
The proctosigmoidectomy resection specimen including the intact mesorectum. 1 - Proximal end of the resection (sigmoid colon). 2 - Rectosigmoid junction. 3 - Distal end of the resection (anus and anal canal). * - Mesorectal fascia.

After approximately three months (during which neoadjuvant treatment was administered), the patient withstood the surgical treatment. This included the abdominoperineal proctosigmoidectomy surgery with the creation of a terminal colostomy. The rectal resection included the total excision of the mesorectum, as shown in Figure 1, by following the avascular "holy plane" of Heald [7]. This was done to respect the principles of oncological radicality, as it has been proven that the lymph nodes draining from the rectum are distributed in a random fashion inside the mesorectum and are not always obvious or palpable [8].

**Figure 2:**
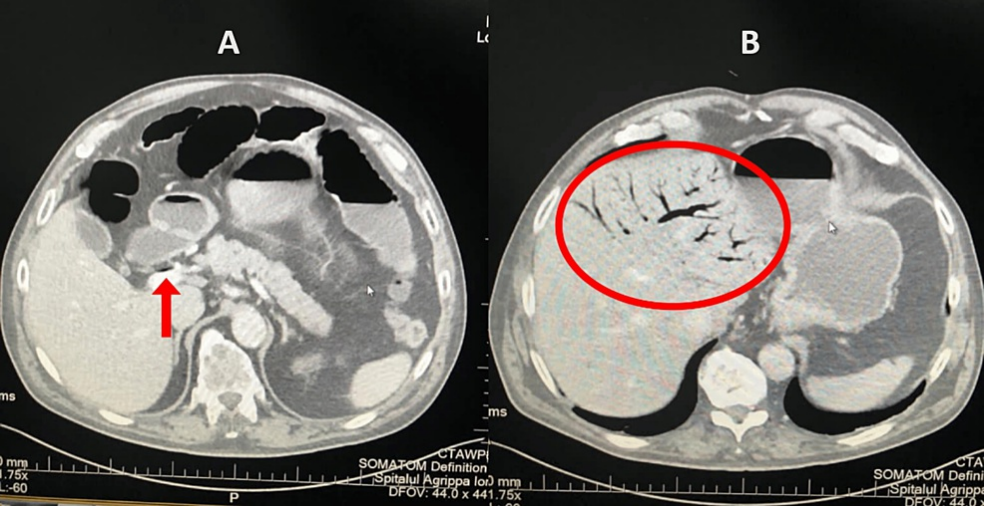
Abdominal/pelvic CT scan with contrast. (A) The arrow points to gas located inside the portal vein. (B) Cranially, gas can be seen inside the branches of the portal vein and inside the liver (circled part).

The postoperative histopathological examination of the resected segment identified the specimen as a rectal mucinous adenocarcinoma, classified as G3, pT3, pN1b, R0, and all the resection margins were clear of tumoral cells. The procedure was followed by a slow but favorable evolution under supportive therapy, during which the patient started to recover and gradually regained his gastrointestinal motility. A few days later, though, the transit for both feces and gas ceased, and the attempts to treat the paralytic ileus (e.g., administration of lactulose and administration of enemas) achieved no positive result. Then, on the 11th postoperative day, following the occurrence of the paralytic ileus, the patient developed an acute episode of HPVG, which was first expressed clinically as acute abdominal pain, which was not remitted following the administration of analgesic drugs. The HPVG was later objectively diagnosed, as the patient was further investigated via ultrasound and CT examinations. Figure 2 represents transversal views of the abdominal/pelvic CT scan, which shows extensive gas collections, branching in accordance with the ramifications of the portal venous system. Figure 2A shows air inside the portal vein before its bifurcation, whereas in Figure 2B, air inside the left ramifications of the portal vein can be observed at the level of the left hepatic lobe.

**Figure 3:**
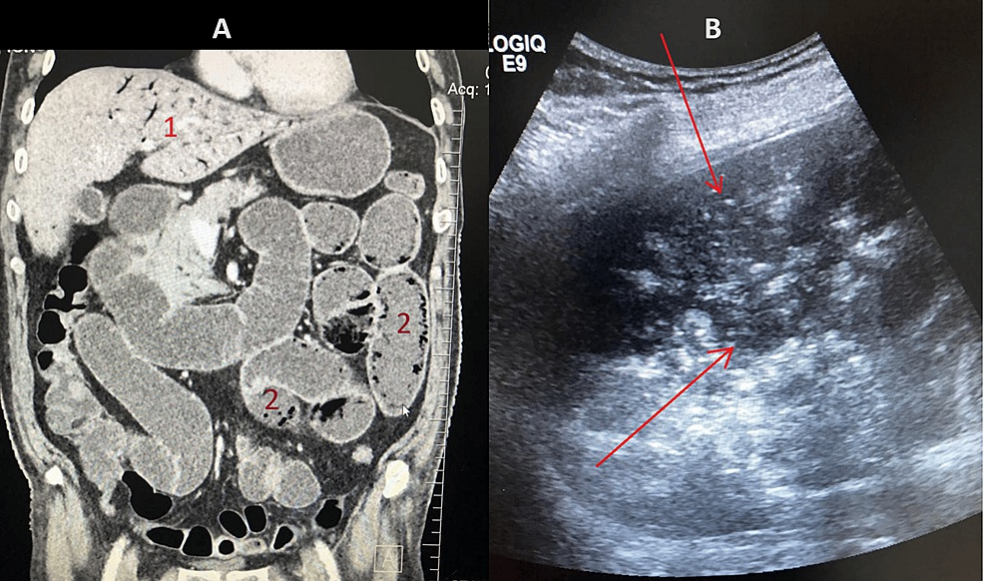
Abdominal/pelvic CT and ultrasonography showing signs of HPVG and small bowel pneumatosis. (A) CT in the frontal plane. 1 - HPVG at the level of the left hepatic lobe. 2 - Jejunoileal pneumatosis. (B) Ultrasound imagery showing multiple portal echogenic foci, indicated by the red arrows. HPVG: hepatic portal venous gas.

In a coronal view of the same scan, shown in Figure 3A, small bowel pneumatosis is demonstrated inside the walls of the jejunal and ileal loops. Hepatic portal venous gas can be seen at the level of the left lobe of the liver, inside the branches of the portal vein. In Figure 3B, which is an abdominal ultrasound examination, a gaseous substance can be identified in the peripheric region of the liver, mainly inside the lumen of the branches of the portal vein.

**Figure 4:**
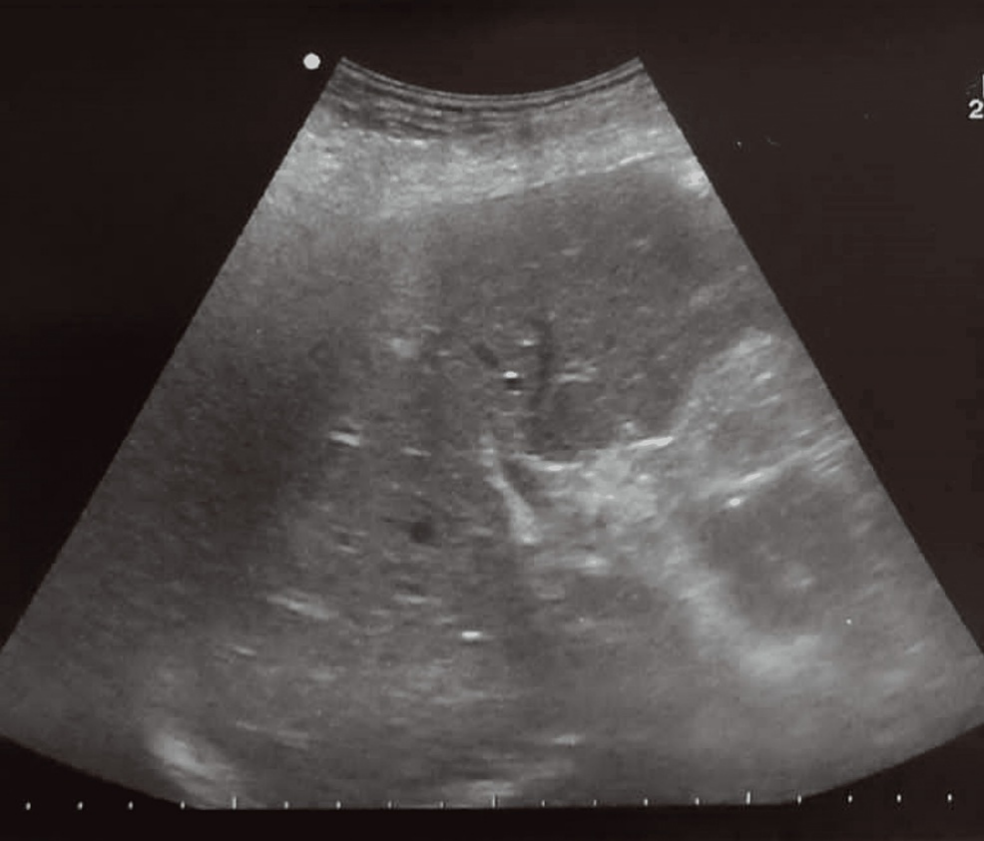
Follow-up ultrasound examination showing complete remission of the hepatic portal venous gas.

The patient was promptly taken to the operating room and underwent an exploratory laparotomy. Intraoperatively, no other causative factors of obstruction (e.g., tumoral mass, extrinsic compression, and anastomotic fistula) or ischemia were observed. An ileostomy was performed to prevent intestinal occlusion. The follow-up ultrasound examination showed the complete resolution of the episode, as seen in Figure 4. The patient was stabilized and received supportive therapy, which greatly improved his clinical status. He was later discharged in favorable condition and was directed to the oncology department to undergo adjuvant chemotherapy.

Approximately half a month after the surgery, the patient returned to the hospital presenting a suture granuloma, which was surgically excised, and then later developed a wound dehiscence, which was treated with silver dressings, leading to complete healing [9].

Approximately two months later, the patient presented once again to the hospital, with clinical and paraclinical findings that showed malabsorption, malaise, and anuria, all of which occurred due to an intestinal evisceration complicated by bowel obstruction. His blood analyses showed elevated C-reactive protein with leucocytosis, anemia, hyperglycemia, hyponatremia, and acute kidney failure (glomerular filtration rate = 14 mL/min/1.73 m2) [10]. The patient underwent another surgery to treat the evisceration and the obstructive syndrome. The evolution of the patient was favorable until five days postoperatively when he developed an enteric fistula at the site of the anastomosis, which was promptly surgically cured. A thoracic radiography and a cerebral contrast CT were also performed to investigate the possible metastatic extension of the initial tumoral process. Neither of them revealed any suspicious cerebral, osseous, intra-abdominal, or intra-thoracic lesions. The patient presented a favorable evolution and was later discharged as surgically cured.

## Discussion

In our patient, a particular aspect is represented by the fact that the episode occurred after paralytic ileus (the most probable cause of HPVG in this case) was diagnosed. The likely consequences of the paralytic ileus, which ultimately led to the passage of gas into the portomesenteric system, include intestinal wall ischemia, accumulation of gas inside the gastrointestinal lumen with the increase of the intraluminal pressure, and possibly the excessive microbial colonization of the small bowel.

Another relatively unusual detail is the quickly favorable evolution of the patient, consecutive to the surgical cure of the HPVG. Early active mobilization of the patient was achieved on the second postoperative day, which is likely to have greatly contributed to a better recovery following the surgery.

Finally, another distinctive feature of our case is given by the long-term survival of the patient after the episode of HPVG, although the mortality rate of this condition is still regarded as rather a very high one. It is highly likely that the complete excision of the mesorectum also contributed greatly to the long-term survival of the patient, as it helped achieve the oncological radicality of the surgery.

HPVG still represents a seriously life-threatening condition, despite the modern advances in terms of diagnosis and treatment possibilities. A thorough analysis of this pathology and meticulous examination of the paraclinical investigations are crucial for a better understanding of how the determining factors lead to the entrance of gas in the capillaries tributary to the portal vein.

Generally, HPVG is considered to be associated with four intertwining causes: sepsis, parietal or mucosal damage, inflammation of the intraperitoneal organs, and meteorism (accumulation of gas inside the GI tract) [11]. These may be found in a variety of pathologies, such as bowel necrosis, abscesses, inflammatory bowel disease, gastric/duodenal ulcer, iatrogenic endoscopic perforations, tumors, and inflammation of the liver, biliary tract, or the pancreas [3]. It is also considered that the presence of bowel necrosis determines a higher chance of developing HPVG [12]. However, rare occurrences, such as this episode that happened because of paralytic ileus, show us that HPVG’s pathogenesis is yet to be completely understood.

One of the factors that contribute to the severity of this complication is represented by the pre-existing terrain on which the HPVG occurs. Another one is the time that passes between the patient coming to the hospital and the doctors obtaining the diagnosis, devising a treatment plan, and ultimately applying it.

Among the essential aspects that may reduce the mortality in cases of HPVG, perhaps the most important one, is a correct and timely diagnosis. Clinically, the patients typically complain of symptoms that are rather general, such as abdominal pain in various degrees or dyspnea [13]. Because the clinical manifestations are typically non-specific to this pathology, the diagnosis is usually done via imagistic examinations, such as point-of-care ultrasound (POCUS), CT, or plain abdominal radiography [14,15]. Because of the generally higher sensitivity, CT is the recommended investigation when suspecting HPVG, if available [16]. When smaller quantities of gas enter the portal venous territory, though, the ultrasound examination is regarded as the better option [17]. Considerable attention must be paid to the differential diagnosis of HPVG with pneumobilia (PB) when the air has already reached the liver [18]. The main difference is that the former is indicated by the peripheric location of the air emboli inside the liver, close to the capsule of the liver (Glisson capsule), due to the centrifugal trajectory of the branches of the portal vein, whereas the latter presents itself as air emboli located further inside the liver, according to the centripetal direction of the biliary ducts [3]. Thanks to the increasingly wider use of CT as a routine diagnostic tool, milder cases of HPVG (i.e., either not requiring surgical treatment or determined by factors of a lesser gravity) have been diagnosed [19,20].

The management of HPVG can follow mainly two directions depending on the patient's clinical status, paraclinical analyses, and, most of all, imagistic findings: the surgical approach or the conservative approach, associated with additional imagistic studies. Nelson et al. proposed an algorithm that recommends an "aggressive" surgical approach in case of HPVG visible on abdominal radiograph or suspicion of bowel ischemia, observation, and further investigation in case of correctable pathologies, such as ulcers and abscesses, and a strictly conservative approach for patients with a history of intestinal pathologies, such as irritable bowel disease, diverticulitis, or fistulae. Though this recommended algorithm may well be applied in most of the cases, each patient should be properly evaluated and the attending surgeon should also rely on their experience with hepatopancreatic and intestinal surgery when deciding on the optimal treatment plan. Thus, the potential benefits of the surgery have to be held against the inherent risks that it poses to the patient. Reversely, it has to be decided if the occurrence of HPVG inflicts more harm to the patient than potentially curative surgery does. Thus, we agree with Daneshmand et al. that if an acute abdomen is excluded during the differential diagnosis, the conservative management of the patient can be not only feasible but the better option for the patient [21].

## Conclusions

Although our patient presented multiple potential causes that could have led eventually to the development of the HPVG episode (e.g., extensive surgery after rectal carcinoma with low anterior resection of the rectum and total mesorectal excision, bowel ischemia, etc.), the most probable cause, in this case, was actually the occurrence of the paralytic ileus. The course of treatment is an aspect that the attending physician should consider carefully. Although, in many cases, surgery may prove to be life-saving, it has to be analyzed opposite to the conservative approach. The biological status of the patient has to be taken into account to assess if their body can better tolerate surgery or the persistence of the HPVG itself.

Despite all the progress we have seen in the last decades regarding surgical technique, medical protocols, and diagnostic technology, HPVG still remains a highly fatal condition, which requires a multidisciplinary approach. Imagistic investigations are the most helpful asset during the diagnostic process and should be used as soon as they are available, so as to shorten the time between the occurrence of the pathology and the initiation of the treatment plan.
